# Exome Analysis of Two Limb-Girdle Muscular Dystrophy Families: Mutations Identified and Challenges Encountered

**DOI:** 10.1371/journal.pone.0048864

**Published:** 2012-11-14

**Authors:** Kristin K. McDonald, Jeffrey Stajich, Colette Blach, Allison E. Ashley-Koch, Michael A. Hauser

**Affiliations:** 1 Center for Human Genetics, Duke University, Durham, North Carolina, United States; 2 Department of Medicine, Duke University Medical Center, Durham, North Carolina, United States; Charité Universitätsmedizin Berlin, NeuroCure Clinical Research Center, Germany

## Abstract

The molecular diagnosis of muscle disorders is challenging: genetic heterogeneity (>100 causal genes for skeletal and cardiac muscle disease) precludes exhaustive clinical testing, prioritizing sequencing of specific genes is difficult due to the similarity of clinical presentation, and the number of variants returned through exome sequencing can make the identification of the disease-causing variant difficult. We have filtered variants found through exome sequencing by prioritizing variants in genes known to be involved in muscle disease while examining the quality and depth of coverage of those genes. We ascertained two families with autosomal dominant limb-girdle muscular dystrophy of unknown etiology. To identify the causal mutations in these families, we performed exome sequencing on five affected individuals using the Agilent SureSelect Human All Exon 50 Mb kit and the Illumina HiSeq 2000 (2×100 bp). We identified causative mutations in *desmin* (IVS3+3A>G) and filamin C (p.W2710X), and augmented the phenotype data for individuals with muscular dystrophy due to these mutations. We also discuss challenges encountered due to depth of coverage variability at specific sites and the annotation of a functionally proven splice site variant as an intronic variant.

## Introduction

Muscular dystrophies and cardiomyopathies are devastating diseases for which no cures or preventative treatments are currently available. There has been great progress in the identification of genetic mutations that cause some forms of muscle disease; however, genetic heterogeneity is the rule rather than the exception. Molecular diagnosis of these disorders is challenging because the large number of known causative genes (more than 100) makes exhaustive clinical testing very expensive and the similarity of clinical presentation makes selection of likely candidate genes difficult [1]. Several strategies have successfully identified mutations causing muscle disorders including sequencing individual candidate genes, homozygosity mapping, linkage analysis and high-throughput screening using a candidate gene panel, whole exome capture, and whole genome sequencing [2], [3], [4].

Whole exome capture and sequencing is relatively cheap and allows investigators to screen many of the known causative genes at one time. Additionally, many orphan dystrophies remain to be characterized, and an advantage of exome sequencing is that it provides information about loci that are not currently known to be associated with muscle disease [1], [5]. Therefore, when a known causative mutation is not identified for a family, researchers have the opportunity to examine novel candidate variants using the same data. As this is a relatively new and continually developing technology, there are some challenges associated with the analysis of this data. Exome capture probes are not able to capture each exon in every gene. Even within regions that contain probes, coverage of insufficient depth or quality can cause a disease-causing variant to remain undiscovered [6]. These regions that lack coverage can vary across individual captures [7], [8]. Annotation of some regions can be inaccurate or incomplete [9]. It is also necessary to perform Sanger sequencing to confirm that an identified variant is present and not a sequencing error [6].

We performed exome sequencing on affected individuals from two families with autosomal dominantly inherited forms of limb-girdle muscular dystrophy to identify disease-causing mutations and to examine the extent of high quality sequence coverage of the known causative genes. We have identified the causative mutations for both families and provided genotype/phenotype correlations. We also discuss specific challenges encountered in the use of this technology.

## Methods

### Ethics Statement

This study adhered to the tenets of the Declaration of Helsinki. Subjects for this study were recruited at Duke University. Participants were informed of the nature and risks of the study, and signed consent forms were obtained. The institutional review board of Duke University Medical Center (DUMC) reviewed and approved this study (protocol Pro00016428).

### Sample Ascertainment

**Figure 1:**
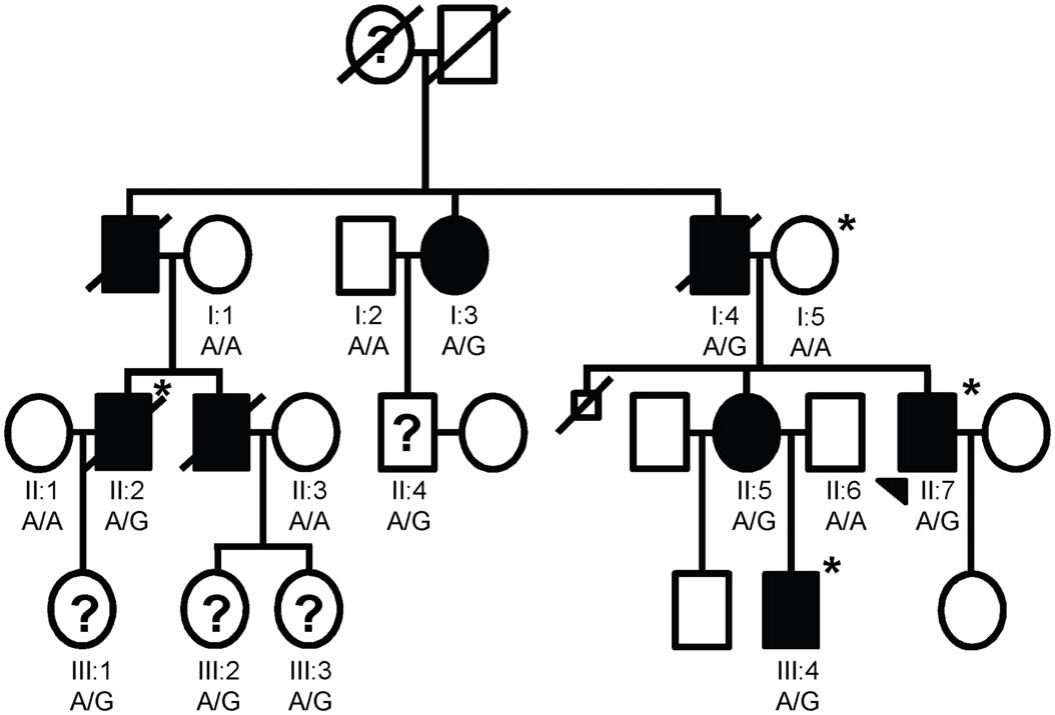
LGMD2359 pedigree and results of Sanger sequencing for *DES* IVS3+3A>G. The pedigree shows the affection statuses, individual identifiers, and genotypes at *DES* IVS3+3. The genotypes obtained through Sanger sequencing of individuals with available high quality DNA are shown. The arrow indicates the proband. * indicates that DNA was sent for exome sequencing. Individuals with ? were of uncertain affection status.

**Figure 2:**
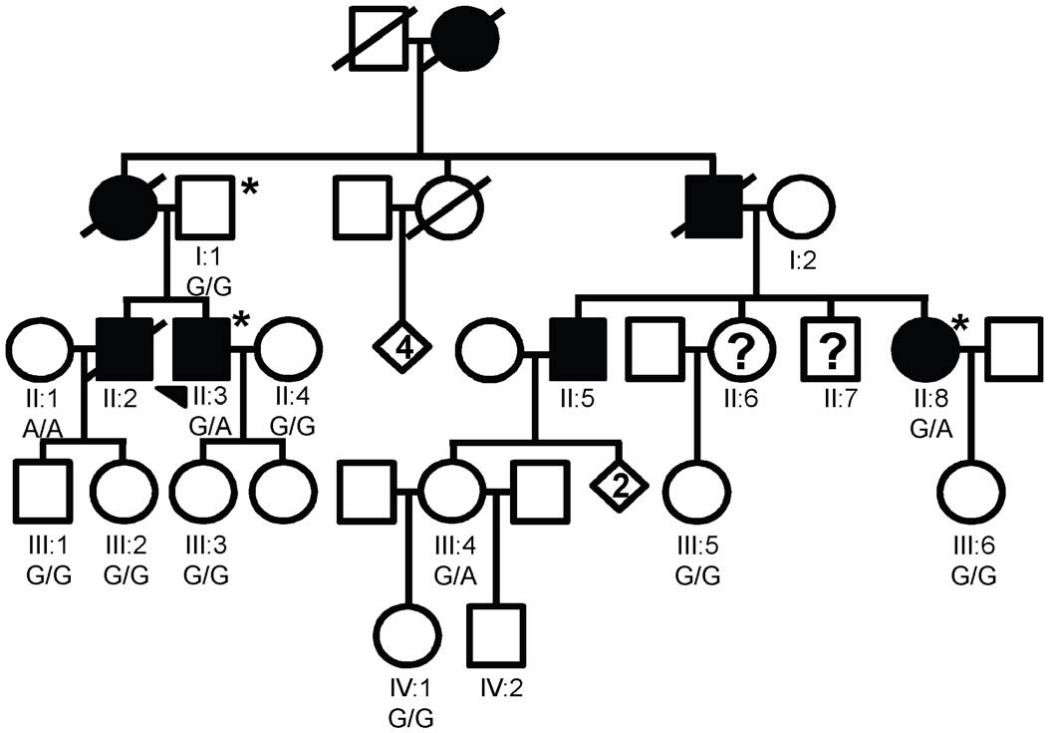
LGMD2692 pedigree and results of Sanger sequencing for FLNC p.W2710X. The pedigree shows the affection statuses, individual identifiers, and genotypes at FLNC p.2710. The genotypes obtained through Sanger sequencing of individuals with available high quality DNA are shown. The arrow indicates the proband. * indicates that DNA was sent for exome sequencing. Individuals with ? were of uncertain affection status.

DNA samples from a total of six affected individuals, four individuals of uncertain affection status, and six unaffected individuals (all are married-in spouses) were collected from family LGMD2359 (Figure 1). Similarly, DNA samples were collected from a total of two affected individuals and ten unaffected individuals from family LGMD2692 (Figure 2). Exome sequencing was performed for three affected individuals in LGMD2359 and two affected individuals in LGMD2692. In addition, one married-in spouse from each family and one unrelated individual who did not show any signs of muscle disease were submitted for exome sequencing as controls. The individuals for whom exome sequencing was performed are indicated by asterisks in Figures 1 and 2. Each sampled individual was Caucasian. Genomic DNA was extracted from blood obtained through venipuncture of participating individuals (Gentra, Minneapolis, MN).

### Exome Capture and Sequencing

Three micrograms of DNA from each individual were submitted to the Center for Human Genome Variation at Duke University for exome sequence capture and amplification using the Agilent SureSelect Human All Exon 50 Mb kit. Sequencing was performed with paired-end 100 base reads on the Illumina HiSeq 2000 platform.

### Alignment, Variant Calling, and Annotation

Alignment of fastq sequence files against the human reference sequence (hg19) and read pairing were performed with the Burrows-Wheeler Aligner v.0.5.6 (BWA) [10]. The SAM files were converted to BAM files and unmapped reads were removed with Samtools v.0.1.7 [11]. File sorting, marking and removal of duplicates, and indexing were performed with Picard v.1.14 (http://picard.sourceforge.net). The resulting file was realigned and recalibrated with the Genome Analysis Tookit v.1.0.5974 (GATK) [12]. Default settings were applied for BWA, Samtools, Picard, and the GATK. Variants were called separately for SNPs and indels using the GATK Unified Genotyper [13] within a 200 bp window surrounding the targeted intervals for the capture kit. Multi-sample calling was performed separately for the two families; the three cases in LGMD2359 were called together while both cases in LGMD2692 were called together. The three control exomes were run through Unified Genotyper individually for SNP calling, but they were included in the Unified Genotyper indel runs for each family. The non-default settings for SNP and indel calling included: minimum base quality (17), minimum mapping quality (20), stand_call_conf (50), stand_emit_conf (10), and dcov (1000). Variants were further annotated using SeattleSeq version 131 (http://snp.gs.washington.edu/SeattleSeqAnnotation131/).

### Assessment of Exome Quality

Summary data for exome quality is provided in Table S1 and was completed as follows.

The format of the fastq files was validated using FastQValidator (http://genome.sph.umich.edu/wiki/FastQValidator). Each BAM file was run through BamValidator (http://genome.sph.umich.edu/wiki/BamValidator) to determine if any syntactic or format violations were present. BamValidator was also used to determine the mapping rate and duplicate rate for each sample.

Sample identity was confirmed after sequencing by comparing SNP calls from previous genotyping on the Illumina GoldenGate Linkage IV Panel to polymorphisms derived from the exome data. Depth of coverage across the expected Agilent SureSelect Human All Exon 50 Mb capture regions was determined using the GATK Depth of Coverage v3.0 (http://www.broadinstitute.org/gsa/wiki/index.php/Depth_of_Coverage_v3.0) under default conditions.

Further analysis of the quality of each exome was performed with Picard (http://picard.sourceforge.net). CalculateHsMetrics was used to calculate both the number and percent of Illumina filter-passing bases that mapped on or near a baited region of the genome for the realigned, recalibrated BAM with unmapped reads and duplicates removed. CollectMultipleMetrics was used to determine the mean insert size for each exome.

### Variant Filtration

Variants were filtered using two different methods: examination of variants within a list of genes known to be involved in muscular dystrophy and/or cardiomyopathy (Table S2) and with an unbiased exome-wide approach. The known muscle disease gene set included genes used in a previous candidate gene list created by Dias et al as well as additional genes found through a literature search [14]. Variant filtering was performed using PL/SQL Developer v8.0 (Allround Automations, Enschede, the Netherlands).

#### Filtering within candidate genes

Variants found in any of the three control exomes were removed from the list of possible causal variants identified in the affected individuals. The purpose of this was to remove false positives resulting from common sequencing and/or processing artifacts. Since both families had affected females and exhibited male to male disease transmission, all variants found on either the X or Y chromosomes were also removed. Variants were also removed if they were found at a frequency of ≥1% in the 1000 genomes European dataset (ftp://ftp.1000genomes.ebi.ac.uk/vol1/ftp/release/20100804/supporting/EUR.2of4intersection_allele_freq.20100804.sites.vcf.gz) and/or in the HapMap Caucasian dataset (ftp://ftp.ncbi.nih.gov/snp/organisms/human_9606/VCF/v4.0/ByPopulationNoGeno/). This strategy would be inappropriate for an autosomal recessive disease due to the risk of removing disease alleles found at a low frequency in the general population due to their presence in unaffected carrier individuals. The list was further restricted to only include SNVs and indels with at least one of the following SeattleSeq 131 annotation terms: nonsense, stop-gain, stop-lost, frameshift, missense, within 10 bp of a distancetosplice (≤10 bp), or coding indels. Finally, any variants that were not shared in all cases within a family were removed. A potential consequence of removing these variants is that it is possible that a mutation would not be identified in both individuals due to a lack of coverage at that position or a sequencing error. Therefore, to avoid missing known pathogenic variants, a literature search was performed to determine whether any variant identified in at least one case has been shown to cause muscle disease.

#### Exome-wide filtering

Variants were filtered as above, but they were not restricted to a list of candidate genes.

### Sanger Sequencing

The primers to confirm the segregation of variants in *desmin (DES)*, *laminin alpha 2 (LAMA2)*, and the DNAJ/HSP40 homolog *DNAJB6* were GCTGCAGGAGGAGATTCAGT and ACCTGCTGTTCCTGAAGCTG, AAACAATGGAAGCCTATGTGAG and TTAGCTGGTTCTGGCAATCC, and CCCTCACACATGCATTTTCTT and CCAGCATTCATGCACAACTA, respectively. These primers were designed using Primer3Plus [15]. Previously published primers were used for PCR and sequencing of the region surrounding the variant in FLNC [16]. Sequencing was performed by Eton Bioscience, Inc (Research Triangle Park, NC).

### Depth of Coverage and Quality Analysis of Bases within Known Muscular Dystrophy Genes

The exon positions for the consensus coding sequences (CCDS) were downloaded from NCBI on September 7^th^, 2011. “Withdrawn” CCDS exons were removed. Target intervals were created that spanned the coding and splice regions of the 102 skeletal and/or cardiac muscle disease genes partially selected from a list of genes created by Dias et al and shown in Table S2 [14]. Splicing regions were defined as within 10 bases of each CCDS exon. GATK Unified Genotyper was run across these intervals using the options Emit_All_Confident_Sites and –glm (BOTH) with dbSNP132 and hg19 as references. The settings that were applied to define confident sites were minimum base quality (17), minimum mapping quality (20), stand_call_conf (50), stand_emit_conf (0), and dcov (1000).

## Results

### 

#### Phenotypes of affected individuals

Clinical descriptors for the affected and questionable individuals in each family are provided in Table 1.

**Table 1:** Clinical description of individuals with skeletal muscle weakness and/or elevated creatine kinase in LGMD2359 and LGMD2692.

Family/Individual	Age onset/AP	CardiacInvolvement?	CK	WeaknessDistribution	Additional Features
LGMD2359/I:3	37/34	Yes	NL	NF, PU = DU, PL<DL	Hearing loss, PN (previous diagnosis of CMT)
LGMD2359/I:4	11/42	Yes	NL	NF, PU<DU, PL = DL	PC, PN (CMT-like symptoms)
LGMD2359/II:2	26/26	Yes	NL	PL = DL	
LGMD2359/II:4*	28/NA	No	NL	NF, PU, PL = DL	
LGMD2359/II:5	26/26	Yes	NL	NF,PU, PL>DL	PC
LGMD2359/II:7	22/19	Yes	NL	NF, PL>DL	THC, PC
LGMD2359/III:1*	11/NA	No	NL	NF, PU = DU, PL = DL	
LGMD2359/III:2*	15/NA	No	NL	NF, PU	
LGMD2359/III:3*	19/NA	No	NL	NF, PU = DU, PL = DL	Scoliosis
LGMD2359/III:4	17/NA	No	1.5	PL = DL	
LGMD2692/II:3	32/NA	No	2.4	PU, PL, DL	Respiratory arrest, on ventilator
LGMD2692/II:2∧	45/NA	No	2.7	PL = DL>>PU	Only deltoid weakness in UE
LGMD2692/II:5∧	46/NA	No	7.7	PL = DL	Weakness in left arm, possibly due to an accident
LGMD2692/II:6* ∧	NA/NA	No	2.1	no weakness	43 at last exam, only symptom elevated CK
LGMD2692/II:7* ∧	NA/NA	No	1.8	no weakness	54 at last exam, only symptom elevated CK
LGMD2692/II:8	51/NA	No	4.0	PL>PU	Biceps weakness in UE; iliopsoas weakness in LE

Age onset is for skeletal muscle weakness NF = neck flexor THC = tight heel cords.

AP = age at pacemaker insertion PU = proximal upper extremities PC = pes cavus.

* = questionable affection status DU = distal upper extremities PN = peripheral neuropathy.

∧ = DNA unavailable for Sanger PL = proximal lower extremities LE = lower extremity.

NL = normal DL = distal lower extremities UE = upper extremity.

CK = reported as ‘times upper limit of normal’ by gender (when abnormal).

### LGMD2359

The pedigree is shown in Figure 1. Ten individuals exhibited a symmetric, limb-girdle pattern of weakness. Almost all also had neck flexor weakness. Five affected individuals have pacemakers due to cardiac abnormalities. The five individuals with skeletal muscle weakness but without cardiac abnormalities are below the oldest age of cardiac disease onset in the family. I:3 and I:4 were initially diagnosed with Charcot-Marie-Tooth disease (CMT) because both patients exhibited peripheral neuropathy, and I:4 had pes cavus. Pes cavus was also seen in two other affected individuals in the family, II:5 and II:7. Other symptoms that are potentially related to disease pathogenesis include scoliosis in III:3, early hearing loss in I:3, and tight heel cords in II:7. No members of the family have presented with elevated serum creatine kinase (CK).

#### Filtering within candidate genes LGMD2359

**Figure 3:**
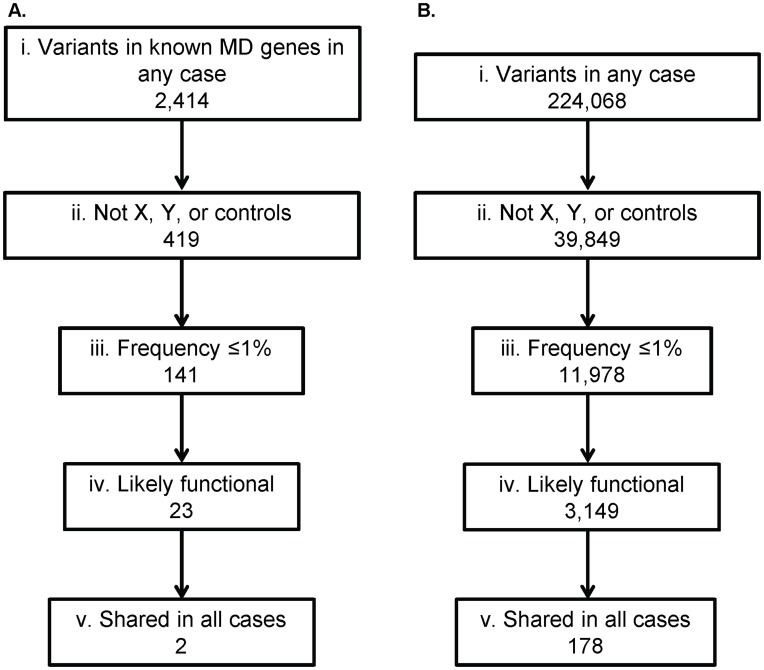
Filtering of variants identified through exome sequencing of individuals in LGMD2359. Variant filtration was performed for all variants (SNVs and indels) identified in known muscle disease genes (A) and in all genes (B). The total number of variants identified in any of the three cases (i) was restricted to variants that were not on the X or Y chromosomes and were not identified in any of the control individuals (ii). This list was further reduced to variants at ≤1% frequency in the HapMap and 1000 Genomes Caucasian databases (iii), then to likely functional variants or variants within 10 bp of splice site (iv), and finally to variants that are shared in all 3 cases (v).

2,004 SNVs and 410 indels located within the 102 muscular dystrophy and/or cardiomyopathy genes were found in at least one of the three cases (Table S2) (Figure 3A). These variants were reduced to 397 SNVs and 22 indels by filtering to remove any present on the X or Y chromosome or present in any of the three control exomes. 141 total variants remained after further removing variants that were found at a frequency of ≥1% in the 1000 genomes European dataset and/or the HapMap Caucasian dataset. This list was restricted to 23 “likely functional or within 10 bases of a splice site” SNVs and indels with at least one of the following SeattleSeq131 annotation terms: nonsense, stop-gain, stop-lost, frameshift, missense, distancetosplice (≤10 bp), or coding indels. A literature search was performed to determine whether these 23 variants are known to be benign or causative (Table S3). Two of these variants are found in all three affected individuals. Sanger sequencing of the entire family showed that the missense variant Y544D in LAMA2 was a false positive from the exome sequencing data. However, the variant in *desmin,* IVS3+3A>G (build37, chr2∶220285071) did segregate with affection status in the family as shown in Figure 1.

The same filtering steps were performed using SNVs and indels found in any gene in the exome to determine how many candidate variants would be present after each step if the gene list was not restricted to a set of candidate genes (Figure 3B). 183,491 SNVs and 40,577 indels were found in at least one case. 11,978 total SNVs and indels were not on either the X or Y chromosome, in any control individuals, or found at a frequency of ≥1% in the 1000 genomes European dataset and/or the HapMap Caucasian dataset. Of those variants, 3,149 were likely functional or within 10 bases of a splice site, and 178 of those were shared in all 3 cases.

### LGMD2692

The pedigree is shown in Figure 2. All four affected individuals presented with proximal weakness in the lower extremities. Three of the four also had weakness in the proximal upper extremities (II:2, II:3, and II:8), while three individuals had weakness in the distal lower extremities (II:2, II:3, and II:5). Muscle weakness was symmetric in all affected individuals with the exception of II:5, who had weakness in the left arm due to an injury. The proband, II:3, suffered respiratory failure at age 55 and now uses a ventilator. Elevated serum CK was observed in all affected individuals. The family is of German ancestry.

#### Filtering within candidate genes LGMD2692

**Figure 4:**
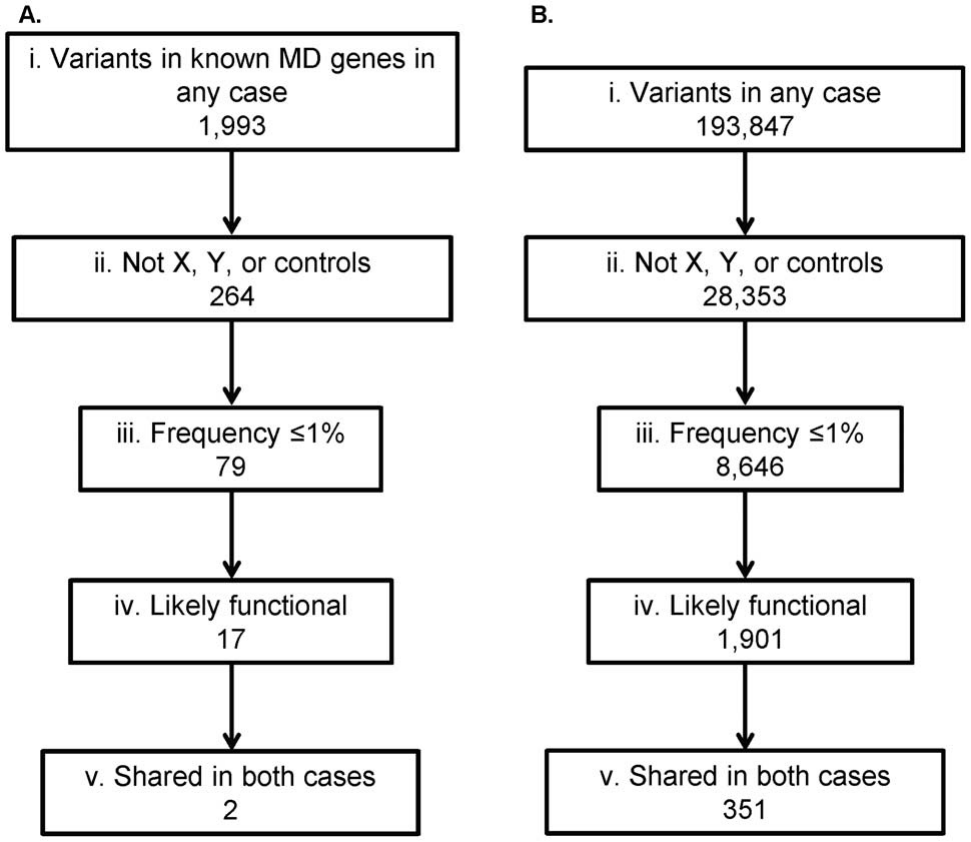
Filtering of variants identified through exome sequencing of individuals in LGMD2692. Variant filtration was performed for all variants (SNVs and indels) identified in known muscle disease genes (A) and in all genes (B). The total number of variants identified in either of the cases (i) was restricted to variants that were not on the X or Y chromosomes and were not identified in any of the control individuals (ii). This list was further reduced to variants at ≤1% frequency in the HapMap and 1000 Genomes Caucasian databases (iii), then to likely functional variants or variants within 10 bases of a splice site (iv), and finally to variants that are shared in both cases (v).

1,641 SNVs and 352 indels were identified in the 102 muscular dystrophy and/or cardiomyopathy genes in at least one of the two cases (Table S2) (Figure 4A). Of these variants, 248 SNVs and 16 indels were not on the X and/or Y chromosomes and were not present in any of the three control exomes. 79 total SNVs and indels remained after removing variants that were found at a frequency of ≥1% in the 1000 genomes European dataset and/or the HapMap Caucasian dataset. The list was further restricted to 18 variants by only including SNVs and indels with at least one of the following SeattleSeq131 annotation terms: nonsense, stop-gain, stop-lost, frameshift, distancetosplice (≤10 bp), missense, or coding indels. Literature searches were performed to determine whether variants found within either of the last two stages of filtering were known to be benign or causative. These variants are shown in Table S4. Two of these variants were found in both affected individuals. Although exome sequencing detected a possible variant in the 3′UTR of the DNAJ/HSP40 homolog *DNAJB6*, we were unable to confirm the presence of the variant via Sanger sequencing because it is located within a homopolymer stretch. However, the variant in FLNC, p.W2710X (build 37, chr7∶128498529), did segregate with affection status in the family as shown in Figure 2. Although unaffected individual III:4 did have the variant, she was only 27 at last exam, which was below the oldest age of onset to date in the family (51).

This filtering was performed with SNVs and indels found in any gene in the exome to show how many candidate variants are present when the gene list was not restricted to a set of candidate genes (Figure 4B). 157,753 SNVs and 36,094 indels were found in at least one case. 8,653 total SNVs and indels were not on either the X or Y chromosome, in any control individuals, or found at a frequency of ≥1% in the 1000 genomes European dataset and/or the HapMap Caucasian dataset. Of those variants, 1,901 were likely functional or within 10 bases of a splice site, and 351 of those were shared in both cases.

#### Depth of coverage analysis

For each exome, positions within the CCDS and splice regions (10 bases 5′ and 3′ of each exon) were identified that did not have high confidence calls and/or adequate read depth to identify heterozygous variants. Any base within these intervals that had <10 filter-passing reads and/or GATK QUAL scores <50 was defined as a “failed” position. 6.5% of bases within the gene set failed in every exome. 3 genes (FKRP, KBTBD13, and PLEC) failed across ≥50% of the CCDS+splice regions in at least one of the affected individuals, and 24 genes failed across ≥30% of the CCDS+splice loci in at least one affected individual. At least 30% of the bases in 8 genes (BIN1, CRYAB, DES, FKRP, KBTBD13, PABPN1, PLEC, and SMN1) were never covered in any of the five exomes from affected individuals. These results, listed in Table 2, show variability in coverage between each of the capture and sequencing runs.

**Table 2:** Known muscle disease genes in which ≥30% of CCDS+splice bases have <10X coverage and/or QUAL scores of <50 in at least one case.

			% CCDS+splice bases failed*					
Gene	# CCDS+splice bases	# CCDS+splice bases failed* all cases	All cases	2359 II:2	2359 II:7	2359 III:4	2692 II:3	2692 II:8
BIN1	2227	672	30.18	52.76	42.30	46.88	48.54	64.08
COL6A1	3787	1106	29.21	42.83	40.22	44.97	37.39	53.45
COL6A2	3962	853	21.53	36.90	35.41	36.19	33.52	46.67
CRYAB	588	223	37.93	37.93	37.93	37.93	37.93	37.93
DES	1593	552	34.65	35.72	38.04	39.80	38.42	40.36
DPM3	389	1	0.26	0.26	1.03	0.51	5.40	33.68
FKRP	1508	1336	88.59	97.35	100.00	95.16	93.04	100.00
GAA	3239	499	15.41	32.94	23.87	28.93	19.76	41.40
JUP	2498	632	25.30	40.07	28.38	36.51	42.31	47.92
KBTBD13	1397	966	69.15	87.54	84.54	80.67	72.01	94.13
LMNA	2256	487	21.59	29.30	25.93	32.80	36.57	66.80
MYBPC3	4505	866	19.22	32.65	27.48	30.17	29.77	43.64
PABPN1	1061	347	32.70	35.63	35.63	44.39	45.33	48.35
PLEC	15508	5934	38.26	51.62	47.47	55.42	42.84	52.12
POMT2	2673	712	26.64	29.55	28.77	26.79	33.56	34.04
SEPN1	2033	560	27.55	28.63	28.14	28.82	27.94	31.23
SGCA	1344	358	26.64	34.38	31.10	35.04	30.80	30.13
SIL1	1566	244	15.58	25.93	22.22	22.73	25.48	34.42
SMN1	1045	975	93.30	93.30	93.30	93.30	93.30	93.30
TAZ	1099	245	22.29	40.58	33.76	30.66	40.22	22.75
TNNI3	793	119	15.01	37.45	24.34	34.68	21.94	50.06
TNNT1	1097	256	23.34	34.46	30.63	26.53	38.38	53.60
TPM1	1650	311	18.85	22.18	26.91	33.88	26.00	25.21
XK	1395	279	20.00	39.43	24.09	30.75	30.39	21.15

* Failed bases are defined as any base with <10 reads at that position and/or a GATK QUAL score of <50.

# CCDS+splice bases = total number of bases in the gene region (consensus coding plus 10 bp on each side for splice sites).

## Discussion

We have evaluated the usefulness of whole exome sequencing as a diagnostic approach for autosomal dominant muscular dystrophy by examining the extent of high quality sequence coverage of known muscle disease genes while searching for disease-causing variants. Exome sequencing was performed to aid in the identification of the disease-causing variants in two families with autosomal dominant forms of muscular dystrophy, LGMD2359 and LGMD2692.

### 

#### LGMD2359 mutation, DES IVS3+3 A>G

Filtering and Sanger sequencing of variants identified through exome sequencing in LGMD2359 affected individuals revealed one variant that is rare, fits the expected inheritance pattern, and is located within a gene known to be involved in muscle disease pathogenesis. The variant alters a base at the splicing position IVS3+3 in *desmin* (*DES*) from an A to a G, and Sanger sequencing shows that it was present in a heterozygous state in four affected individuals as well as in four individuals of questionable disease status. Desmin is an intermediate filament found in striated muscle that forms a three-dimensional scaffold that crosses the myofibril [17]. The filaments surround the Z-disk, associate with the sarcolemma at costameres, and form links to cytoplasmic organelles and the nucleus [18]. Mutations in *DES* disrupt the normal desmin intermediate filament networks and are known to cause a form of myofibrillar myopathy with a limb-girdle phenotype and/or cardiomyopathy [18], [19], [20]. The splicing variant *DES* IVS3+3 A>G results in the excision of the third exon of desmin, aggregate formation, and the disruption of these networks as first shown by Park et al [20], [21]. More recently, Greenberg et al identified this variant as the cause of disease in a family previously associated with the locus LGMD1D [22]. Since the splicing variant has been shown in multiple families with similar phenotypes and functional work has shown that the variant affects protein function, we believe that the mutation *DES* IVS3+3 A>G is responsible for the skeletal and cardiac muscle disease in family LGMD2359. Additionally, we have learned that testing for desmin mutations was recommended to some members of the family; the clinical test identified the *DES* IVS3+3 A>G splicing mutation as well.

Interestingly, because it is not in a canonical splice site, the functional annotation fields in SeattleSeq 131 did not indicate that it is a splicing variant. Use of the “distancetosplice” field for filtering, however, did result in easy identification of the variant although it does include synonymous variants. More recently, SeattleSeq 134 added a “near-splice” annotation that can also be used for filtering splice variants. It is important for researchers to understand how splice sites are defined by the annotation program they use.

#### LGMD2692 mutation, FLNC p.W2710X

Filtration of exome sequencing variants in LGMD2692 resulted in the identification of a nonsense mutation at protein position 2710 in *filamin C* (*FLNC*). The filamins are involved in organizing actin networks and link them to cellular membranes [23], [24]. FLNC p.W2710X is located within the dimerization domain and causes autosomal dominant myofibrillar myopathy with a limb-girdle phenotype. This alteration prevents proper dimerization and increases susceptibility to proteolysis, resulting in the accumulation of cytoplasmic aggregates in the muscles of patients [25], [26]. Family LGMD2692 is of German origin, as are multiple other families with this mutation [27].

#### Challenges of identifying mutations with exome sequencing

While exome sequencing allows many genes to be screened at one time, it results in a large list of variants, including some false positives. When a disease is rare and highly penetrant, the variants can be reduced by restricting the candidate list to those which cause splice site, missense, frameshift, or nonsense changes and removing those that do not fit the expected inheritance pattern in a family or are found at a high frequency in control populations. This method of filtering has been used to successfully identify disease-causing mutations, including recently by Willemsen et al to identify mutations in DYNC1H1 that cause severe intellectual disability with neuronal migration defects and by Daoud et al to identify mutations in SPG11 that cause juvenile amyotrophic lateral sclerosis [28], [29]. These filtering strategies do not focus on the removal of false positive variants, and it can be tempting to limit them by implementing stringent depth and/or quality filters. In some situations, however, researchers may benefit from examining every variant obtained in a candidate gene set without applying these filters. This is the case when a disorder has a large number of candidate genes, and it is cost-prohibitive to screen them with Sanger sequencing prior to exome capture. For example, Sirmaci et al encountered a problem using a minimum depth cut-off to examine potential disease-causing variants found in exomes from individuals with hereditary hearing loss. Using a minimum depth of coverage of 8× as an initial filter, they missed a novel variant in *GIPC3*, a gene which is known to be involved in deafness. If they had not lowered the depth of coverage filter, they may have believed that a different variant in *ZNF57* caused disease [7].

In our study, the p.W2710X variant in FLNC was covered to a reasonable depth of 16 in II:3 in LGMD2692, but there were only 5 reads covering that base in II:8. If a minimum depth cut-off across affected individuals had been applied, the known disease-causing, functionally proven mutation would not have been identified. We were able to restrict our initial candidate list to 23 by requiring that variants be on an autosome, not present in 3 control exomes, at a ≤1% allele frequency in Caucasians from 1000 Genomes and HapMap, annotated as likely functional or near a splice site, and within a candidate gene list. The same filtering without the candidate gene list results in a more difficult to examine list of 3,149 variants in at least one case, and 178 variants that are shared in both cases (Figure 4).

In addition to ensuring that the variants obtained through exome sequencing are thoroughly examined, it is important to determine whether regions of genes that are known to be involved in a given disease are covered with a sufficient depth and quality of reads to confidently discover a variant at that site. Depth of coverage is particularly important when examining an autosomal dominantly inherited disorder because a sufficient number of high-quality reads must be present at a given base to identify two alleles. We investigated the depth and quality of coverage of genes involved in skeletal and cardiac muscle diseases in each of the five exomes from affected members of LGMD2359 and LGMD2692. Target intervals spanned the coding and splice regions (CCDS+splice) of 102 skeletal and/or cardiac muscle disease genes. For each exome, “failed” positions were identified that had <10 reads and/or QUAL scores <50. Similar to the results shown by Dias et al, our data indicate that while many causative genes are well-covered, gaps exist which may interfere with the identification of some disease-causing mutations [14]. While only 6.5% of bases within the gene set failed in every exome, gaps in coverage varied on an individual basis. We found that 24 genes failed across ≥30% of the CCDS+splice loci in at least one affected individual, but only 8 of those genes failed across ≥30% of the CCDS+splice loci in all 5 individuals. In some cases, these gaps may be filled by increasing overall coverage or using a different exome capture method. However, it is likely that other gaps are due to low mapping quality and will not be filled until longer reads are available. Examining exome coverage on an individual basis is useful to determine whether high priority candidate genes were adequately screened.

### Conclusions

In conclusion, we examined the phenotypes of two extended families with limb-girdle muscular dystrophy and used whole exome sequencing data to identify the mutations causing disease, *DES* IVS3+3 A>G and FLNC p.W2710X. While exome sequencing provides reliable, high-quality data for most exons in known muscle disease genes, gaps and low quality regions remain that must be evaluated on an individual basis. Additionally, filtering using distance to splice sites rather than disruption of canonical splice sites avoids potentially missing functional variants.

## Supporting Information

Table S1 **Quality metrics for exomes of affected individuals in LGMD2359 and LGMD2692.** PF = passes Illumina filter # PF bases aligned = the number of bases in the PF aligned reads that are mapped to a reference base. %PF aligned bases on or near bait = On+Near Bait Bases/PF Bases Aligned.
(XLSX)

Table S2 **Set of genes known to be involved in muscle disease used for exome variant filtering and coverage analysis.** * Coverage examined by Dias et al.
(XLSX)

Table S3 **Low frequency variants that are likely functional or near splice sites in known muscle disease genes identified through exome sequencing of LGMD2359 individuals II:2, II:7, and III:4.** GT = genotype (0/0 = homozygous reference, 0/1 = heterozygous) AD = allele depth DP = depth (# reads at variant) QUAL = GATK QUAL score at variant position * = heterozygous for variant in all three affected individuals Variants were considered low frequency if they were found at a frequency of ≤1% in the 1000 genomes European dataset (ftp://ftp.1000genomes.ebi.ac.uk/vol1/ftp/release/20100804/supporting/EUR.2of4intersection_allele_freq.20100804.sites.vcf.gz) and/or in the HapMap Caucasian dataset (ftp://ftp.ncbi.nih.gov/snp/organisms/human_9606/VCF/v4.0/ByPopulationNoGeno/). Near splice site is defined as within 10 bases of a splice site by SeattleSeq 131 (http://snp.gs.washington.edu/SeattleSeqAnnotation131/).
(XLSX)

Table S4 **Low frequency variants that are likely functional or near splice sites and in known muscle disease genes identified through exome sequencing of LGMD2692 individuals II:3 and II:8.** GT = genotype (0/0 = homozygous reference, 0/1 = heterozygous) AD = allele depth DP = depth (# reads at variant) QUAL = GATK QUAL score at variant position * = heterozygous for variant in all three affected individuals Variants were considered low frequency if they were found at a frequency of ≤1% in the 1000 genomes European dataset (ftp://ftp.1000genomes.ebi.ac.uk/vol1/ftp/release/20100804/supporting/EUR.2of4intersection_allele_freq.20100804.sites.vcf.gz) and/or in the HapMap Caucasian dataset (ftp://ftp.ncbi.nih.gov/snp/organisms/human_9606/VCF/v4.0/ByPopulationNoGeno/). Near splice site is defined as within 10 bases of a splice site by SeattleSeq 131 (http://snp.gs.washington.edu/SeattleSeqAnnotation131/).
(XLSX)

## References

[1] Vasli N, Bohm J, Le Gras S, Muller J, Pizot C, et al.. (2012) Next generation sequencing for molecular diagnosis of neuromuscular diseases. Acta Neuropathol. 10.1007/s00401-012-0982-8 PMC3400754 22526018

[2] Boyden SE, Salih MA, Duncan AR, White AJ, Estrella EA, et al (2010) Efficient identification of novel mutations in patients with limb girdle muscular dystrophy. Neurogenetics 11: 449–455. 20623375 10.1007/s10048-010-0250-9 PMC2944962

[3] Malayeri FA, Panjehpour M, Movahedian A, Ghaffarpour M, Zamani GR, et al (2011) Detection of Duchenne/Becker muscular dystrophy carriers in a group of Iranian families by linkage analysis. Acta Med Iran 49: 142–148. 21681700

[4] Wheeler M, Pavlovic A, DeGoma E, Salisbury H, Brown C, et al (2009) A new era in clinical genetic testing for hypertrophic cardiomyopathy. J Cardiovasc Transl Res 2: 381–391. 20559996 10.1007/s12265-009-9139-0

[5] Landstrom AP, Adekola BA, Bos JM, Ommen SR, Ackerman MJ (2011) PLN-encoded phospholamban mutation in a large cohort of hypertrophic cardiomyopathy cases: summary of the literature and implications for genetic testing. Am Heart J 161: 165–171. 21167350 10.1016/j.ahj.2010.08.001 PMC6311091

[6] Ku CS, Cooper DN, Polychronakos C, Naidoo N, Wu M, et al (2012) Exome sequencing: dual role as a discovery and diagnostic tool. Ann Neurol 71: 5–14. 22275248 10.1002/ana.22647

[7] Sirmaci A, Edwards YJ, Akay H, Tekin M (2012) Challenges in whole exome sequencing: an example from hereditary deafness. PLoS One 7: e32000. 22363784 10.1371/journal.pone.0032000 PMC3283682

[8] Kiezun A, Garimella K, Do R, Stitziel NO, Neale BM, et al (2012) Exome sequencing and the genetic basis of complex traits. Nat Genet 44: 623–630. 22641211 10.1038/ng.2303 PMC3727622

[9] Biesecker LG, Shianna KV, Mullikin JC (2011) Exome sequencing: the expert view. Genome Biol 12: 128. 21920051 10.1186/gb-2011-12-9-128 PMC3308041

[10] Li H, Durbin R (2009) Fast and accurate short read alignment with Burrows-Wheeler transform. Bioinformatics 25: 1754–1760. 19451168 10.1093/bioinformatics/btp324 PMC2705234

[11] Li H, Handsaker B, Wysoker A, Fennell T, Ruan J, et al (2009) The Sequence Alignment/Map format and SAMtools. Bioinformatics 25: 2078–2079. 19505943 10.1093/bioinformatics/btp352 PMC2723002

[12] McKenna A, Hanna M, Banks E, Sivachenko A, Cibulskis K, et al (2010) The Genome Analysis Toolkit: a MapReduce framework for analyzing next-generation DNA sequencing data. Genome Res 20: 1297–1303. 20644199 10.1101/gr.107524.110 PMC2928508

[13] DePristo MA, Banks E, Poplin R, Garimella KV, Maguire JR, et al (2011) A framework for variation discovery and genotyping using next-generation DNA sequencing data. Nat Genet 43: 491–498. 21478889 10.1038/ng.806 PMC3083463

[14] Dias C, Sincan M, Cherukuri PF, Rupps R, Huang Y, et al (2012) An analysis of exome sequencing for diagnostic testing of the genes associated with muscle disease and spastic paraplegia. Hum Mutat 33: 614–626. 22311686 10.1002/humu.22032 PMC3329376

[15] Untergasser A, Nijveen H, Rao X, Bisseling T, Geurts R, et al (2007) Primer3Plus, an enhanced web interface to Primer3. Nucleic Acids Res 35: W71–74. 17485472 10.1093/nar/gkm306 PMC1933133

[16] Odgerel Z, van der Ven PF, Furst DO, Goldfarb LG (2010) DNA sequencing errors in molecular diagnostics of filamin myopathy. Clin Chem Lab Med 48: 1409–1414. 20578970 10.1515/CCLM.2010.272

[17] Kouloumenta A, Mavroidis M, Capetanaki Y (2007) Proper perinuclear localization of the TRIM-like protein myospryn requires its binding partner desmin. J Biol Chem 282: 35211–35221. 17872945 10.1074/jbc.M704733200

[18] Capetanaki Y, Bloch RJ, Kouloumenta A, Mavroidis M, Psarras S (2007) Muscle intermediate filaments and their links to membranes and membranous organelles. Exp Cell Res 313: 2063–2076. 17509566 10.1016/j.yexcr.2007.03.033

[19] Goldfarb LG, Park KY, Cervenakova L, Gorokhova S, Lee HS, et al (1998) Missense mutations in desmin associated with familial cardiac and skeletal myopathy. Nat Genet 19: 402–403. 9697706 10.1038/1300

[20] Dalakas MC, Park KY, Semino-Mora C, Lee HS, Sivakumar K, et al (2000) Desmin myopathy, a skeletal myopathy with cardiomyopathy caused by mutations in the desmin gene. N Engl J Med 342: 770–780. 10717012 10.1056/NEJM200003163421104

[21] Park KY, Dalakas MC, Goebel HH, Ferrans VJ, Semino-Mora C, et al (2000) Desmin splice variants causing cardiac and skeletal myopathy. J Med Genet 37: 851–857. 11073539 10.1136/jmg.37.11.851 PMC1734475

[22] Greenberg SA, Salajegheh M, Judge DP, Feldman MW, Kuncl RW, et al (2012) Etiology of limb girdle muscular dystrophy 1D/1E determined by laser capture microdissection proteomics. Ann Neurol 71: 141–145. 22275259 10.1002/ana.22649

[23] Stossel TP, Condeelis J, Cooley L, Hartwig JH, Noegel A, et al (2001) Filamins as integrators of cell mechanics and signalling. Nat Rev Mol Cell Biol 2: 138–145. 11252955 10.1038/35052082

[24] van der Flier A, Sonnenberg A (2001) Structural and functional aspects of filamins. Biochim Biophys Acta 1538: 99–117. 11336782 10.1016/s0167-4889(01)00072-6

[25] Vorgerd M, van der Ven PF, Bruchertseifer V, Lowe T, Kley RA, et al (2005) A mutation in the dimerization domain of filamin c causes a novel type of autosomal dominant myofibrillar myopathy. Am J Hum Genet 77: 297–304. 15929027 10.1086/431959 PMC1224531

[26] Lowe T, Kley RA, van der Ven PF, Himmel M, Huebner A, et al (2007) The pathomechanism of filaminopathy: altered biochemical properties explain the cellular phenotype of a protein aggregation myopathy. Hum Mol Genet 16: 1351–1358. 17412757 10.1093/hmg/ddm085

[27] Kley RA, Hellenbroich Y, van der Ven PF, Furst DO, Huebner A, et al (2007) Clinical and morphological phenotype of the filamin myopathy: a study of 31 German patients. Brain 130: 3250–3264. 18055494 10.1093/brain/awm271

[28] Daoud H, Zhou S, Noreau A, Sabbagh M, Belzil V, et al.. (2012) Exome sequencing reveals SPG11 mutations causing juvenile ALS. Neurobiol Aging 33: 839 e835–839. 10.1016/j.neurobiolaging.2011.11.012 22154821

[29] Willemsen MH, Vissers LE, Willemsen MA, van Bon BW, Kroes T, et al (2012) Mutations in DYNC1H1 cause severe intellectual disability with neuronal migration defects. J Med Genet 49: 179–183. 22368300 10.1136/jmedgenet-2011-100542

